# Comment on Morse et al. A Universal Pharmacokinetic Model for Dexmedetomidine in Children and Adults. *J. Clin. Med.* 2020, *9*, 3480

**DOI:** 10.3390/jcm10143003

**Published:** 2021-07-06

**Authors:** Douglas J. Eleveld, Pieter J. Colin, Laura N. Hannivoort, Anthony R. Absalom, Michel M. R. F. Struys

**Affiliations:** 1 Department of Anesthesiology, University Medical Center Groningen, University of Groningen, 9713 Groningen, The Netherlands; p.j.colin@umcg.nl (P.J.C.); l.hannivoort@umcg.nl (L.N.H.); a.r.absalom@umcg.nl (A.R.A.); m.m.r.f.struys@umcg.nl (M.M.R.F.S.); 2 Department of Basic and Applied Medical Sciences, Ghent University, 10, 9000 Ghent, Belgium

We read with interest the recently published manuscript [1] by Morse, Cortinez and Anderson in which they describe the development of a universal pharmacokinetic model for dexmedetomidine and mentioned its potential use for target-controlled-infusion (TCI). We would like to caution readers that use of the model as published in a TCI system would lead to initial dexmedetomidine doses considerably higher than those recommended in the summary of product characteristics (SmPC). In general, for plasma-targeted TCI, the size of the loading dose is simply calculated as follows:

Loading dose (μg) = V1 (L) × target plasma concentration (ng/mL).

If the Morse model is used for a TCI with an initial target concentration of 1 ng/mL, the V1 of 25.2 L/70 kg will result in a loading dose of 25.2 μg for an individual who weighs 70 kg (the loading dose is thus 0.36 μg/kg). If this loading dose is administered as a rapid infusion (the default for many TCI infusion pumps) then the initial infusion rate will exceed the recommended maximum infusion rate of 6 μg/kg/h (loading dose 1 μg/kg over 10 min) [2] by an order of magnitude. This is an “off-label” loading dose infusion rate. Although there is no published scientific literature on the likely effects of this administration rate, clinical experience suggests that hypertension and bradycardia will occur.

In the discussion, the authors address the V1 from a number of studies in the literature. They note that Hannivoort et al. [3] found a considerably smaller V1 of 1.78 l/70 kg and describe their V1 of 25.2 l/70 kg as “more realistic”. What seems unrecognized is that the “small” V1 of the Hannivoort models plays a positive role in patient safety because it reduces TCI loading doses compared to models with larger V1. This is illustrated in Figure 1, showing a simulation of dexmedetomidine TCI for the Morse (1 ng/mL) and Hannivoort (1 ng/mL and 1.2 ng/mL) models along with dosing ranges recommended by the SmPC for adults (1 μg/kg over 10–15 min, thereafter 0.2–1 μg/kg/h). The Hannivoort model is “naturally” limited close to 6 μg/kg/min for young children, children, adults and obese. In contrast, TCI with the Morse model initially administers a greater than recommended dose more rapidly.

The authors attribute the “small” V1 of the Hannivoort model to fact that her study methodology involved TCI drug administration in combination with early blood sampling and a possible role of vasoconstriction at higher concentrations. However, a comparatively small V1 is not evidence that it is wrongly estimated. During design of the Hannivoort study we recognized the pivotal role of V1 on TCI drug administration, which is why the study methods included a short infusion 10 min before starting TCI. This was found to provide more informative estimates of V1 compared to other designs. In contrast, the study data used by Morse contains mixed arterial and venous samples, slow infusions, and no early sampling, all issues that would tend to bias V1 estimates upwards. We do not think the Hannivoort V1 should be dismissed as “unrealistic” based on its dissimilarity to other studies with less informative designs.

Proposing a PK model for TCI simply based on its ability to predict concentration observations from a PK study takes a myopic view of TCI systems. Sources such as product labels, dosing guidelines, and clinical experience provide essential information about safe and adequate drug administration. These reflect a physiological reality broader than a PK modeling study. TCI systems and the drug administration they provide should be seen from this wider perspective. We think the Hannivoort model provides a safer dexmedetomidine TCI because drug administration is closer to recommendations that the infusion rate should be limited to 6 μg/kg/h. If the Morse model is used in a TCI system it is critical that infusion rates are limited to safe levels.

## Figures and Tables

**Figure 1:**
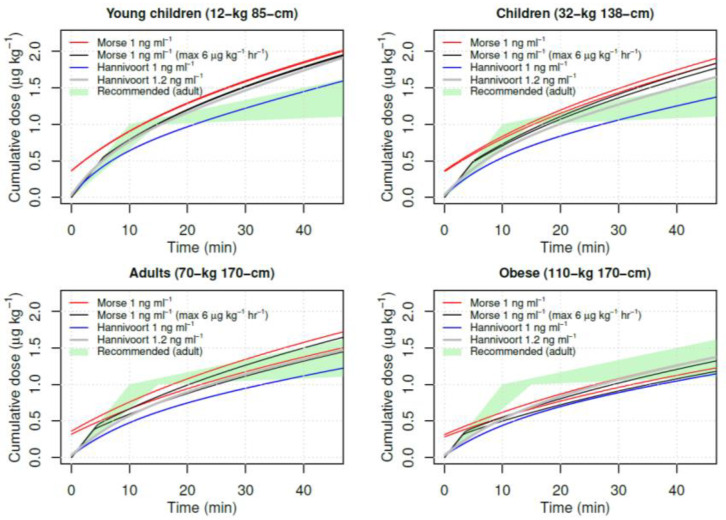
Simulation of dexmedetomidine TCI for the Morse (1 ng/mL) (male and female) and Hannivoort (1 ng/mL and 1.2 ng/mL) models along with recommended dosing ranges in adults (1 μg/kg over 10–15 min, thereafter 0.2–1 μg/kg/h).
